# The Development of Young Peoples’ Internalising and Externalising Difficulties Over the First Three-Years in the Public Care System

**DOI:** 10.1177/10775595211070765

**Published:** 2022-02-01

**Authors:** Rachel M Hiller, Abigail Fraser, Megan Denne, Andreas Bauer, Sarah L Halligan

**Affiliations:** 1 Division of Psychology & Language Sciences, University College London, UK; 2 Anna Freud Centre for Children and Families, UK; 3 Department of Psychology, 1555 University of Bath, UK; 4 Population and Health Sciences, Bristol Medical School, 1980 University of Bristol, UK; 5 Department of Psychiatry and Mental Health, University of Cape Town, South Africa

**Keywords:** internalising, externalising, longitudinal, child maltreatment, child welfare, foster care, out-of-home care

## Abstract

Although we know there are high rates of mental health difficulties amongst young people in out-of-home care (i.e. social welfare-involved children), there is limited evidence on the longitudinal development of these problems, particularly from when they enter the care system. Using the routinely collected carer-reported strengths and difficulties questionnaire, we explored internalising (emotional and peer) and externalising (conduct and hyperactivity) difficulties for 672 young people across their first 3 years in the UK care system (2–16 yrs, 51% boys, 76% Caucasian). In all cases stable profiles (resilient or chronic) were most common, while changing profiles (recovery or delayed) were less common. Findings showed that entry into the care system is not enough of an intervention to expect natural recovery from mental health difficulties. Number of placements and being separated from siblings were associated with greater difficulties. Implications for child welfare and mental health systems are discussed.

Young people who have been removed from their family home and placed in the public out-of-home care system (sometimes called social welfare-involved or looked-after children) represent a particularly vulnerable group of youth. The most common reason a child or teen would be placed in care is to keep them safe from abuse and/or neglect. In the UK, most young people are placed in care from school-age or older, meaning for many, exposure to abuse and/or neglect has been ongoing for many years (Department for Education [Department for Education, 2019]. Child maltreatment is a key predictor of later psychopathology (Keyes et al., 2012), as are the many other forms of adversity that have often been experienced by young people in care (e.g. poverty, parental mental health; Wadman et al., 2020). Once in care, ongoing instability can also be common, including separation from siblings and moves between different caregivers (Department for Education, 2019), which may further exacerbate distress (Newton et al., 2000). Given the accumulation of risk factors, it is perhaps unsurprising that high rates of psychopathology have been well-documented in this group (e.g. Brand & Brinich, 1999; Jozefiak et al., 2016; McAuley & Davis, 2009; Vis et al., 2016). In the UK, a survey of over 1000 young people in care found they were approximately five times more likely to meet criteria for a psychiatric disorder compared to their peers, with elevated rates of both internalising and externalising problems (Ford et al., 2007). A US-study of almost 400 17-year-olds in foster care found 61% met diagnostic criteria for at least one psychiatric disorder (McMillen et al., 2005). Qualitative evidence also continues to highlight the substantial unaddressed psychological needs of this group (e.g. Rock et al., 2013), which can have a long-term influence on their broader wellbeing (Jones et al., 2016; Teyhan et al., 2018).

Although there is growing evidence on the prevalence of mental health difficulties for young people in care, there remains limited evidence of the longitudinal trajectories of these difficulties, particularly from when they enter the child welfare system (see Tarren-Sweeney & Goemans, 2019). Such information is crucial for better understanding the needs of this group, including whether there are critical periods from entering care where intervention or prevention programmes could be targeted to improve the picture of mental health outcomes. Further, understanding whether particular types of difficulties (e.g. emotional and conduct) develop differently or similarly over time is potentially particularly useful information for this group, given evidence that certain types of difficulties (particularly those that are more overt) may be more likely to facilitate mental health service access (Conn et al., 2016).

Mapping how internalising and externalising difficulties may change over time, rather than only presenting the cross-sectional profiles is particularly important for a group who often face ongoing instability. Research (that is largely US-based) has long highlighted associations between ongoing instability of care, deteriorations in carers’ perceived ability to provide support, and high emotional and behavioural difficulties (James et al., 2004a; Lindhiem & Dozier, 2007; Newton et al., 2000; Rock et al., 2013). Although a lack of high-quality longitudinal evidence prevents conclusions on causality, it is generally agreed that these associations are likely reciprocal, with a lack of secure and consistent placement and care worsening a young person’s mental health, and worsening emotional or behavioural difficulties presenting carers with challenges that they might struggle or feel unsupported to manage (Lindhiem & Dozier, 2007; Orme & Buehler, 2001). Research, again from the US, shows that frequent placement changes are associated with high rates of mental health service utilisation (James et al., 2004b; Rubin et al., 2004). There is less evidence to understand how services respond to these acute periods of crisis, or whether there is continuation of care to prevent future breakdowns. In the UK, placement stability can be considered important before accessing a mental health service for therapeutic intervention, meaning those with the greatest mental health needs can be stuck in a cycle where accessing high quality and continued professional mental health support remains elusive (Hiller & St Clair, 2018). Given the changes inherent in the lives of many young people in care, even over relatively short periods of time, understanding how mental health difficulties develop across time remains an important area of investigation to more clearly understand the needs of this group.

In England, local authorities are required to collect routine evidence of the emotional and behavioural needs of their young people in care, via a yearly carer-reported strengths and difficulties questionnaire (SDQ; Goodman, 2001). The SDQ is a widely-used and well-validated assessment of four domains of difficulties: emotional problems, peer problems, conduct problems, and hyperactivity. It also includes a subscale on prosocial skills. Research on the use of SDQs within local authorities has shown that the measure provides relatively good predictions of psychiatric diagnoses (Goodman et al., 2004). The routine collection of this data provides a particularly useful avenue for identifying the longitudinal profiles of internalising and externalising difficulties in this group, from when they first enter care. Drawing on this service data, the aim of this work was to provide evidence for the development of emotional and behavioural difficulties in this group, over their first 3 years in the care system. As described in the Methods, we focused on four potential profiles: (i) resilient (low scores), (ii) chronic difficulties, (iii) recovery and (iv) delayed difficulties. The second aim was to explore whether there were basic predictors of internalising and externalising problems that could guide services in identifying the most at-risk young people, and whether different emotional and behavioural profiles might be associated with key markers of instability for young people in care, including number of placement providers, whether or not they live with siblings, and missing person reports. Although it is generally accepted that poorer mental health is associated with greater instability in care there exists limited quantitative evidence of how longitudinal profiles may be associated with such experiences.

## Method

### Sample

Ethical approval was obtained from the University of Bath Psychology Research Ethics Committee, with further approvals/permissions provided by participating local authorities. Data were collated from three English local authorities, which were: medium sized and urban (*n* = 365); medium sized covering a large urban and rural area (*n* = 308); and smaller sized and urban (Site three; *n =* 69). The research team worked with the local authorities to extract completely anonymised data from the service records of young people who had entered care between 2012 and 2016 and had stayed in the care system for at least 2.5 years (the approximate average time a young person remains in care in the UK; Department for Education, 2019), and thus could have feasibly had 3 years of SDQs completed. Data were extracted based on the young person being under the care of the local authority, irrespective of where the young person was geographically located, including if they were located ‘out of area’. The year 2012 was the lower limit as this is when the local authorities moved to an electronic records system, while 2016 was the upper limit to allow the young person to have been in care for the 3-year period (data were extracted in 2019).

Altogether, completely anonymised data were collated for 742 young people (see Table 1 for descriptives). The sample are young people who had entered these local authorities between 2012 and 2016 and stayed within their care for 2.5 consecutive years, with the exception of a small number of restricted cases where data protection meant they were not able to be accessed even for anonymised data. The young people had entered care between birth and 16 years of age (*M* = 9.86 years old, *SD* = 4.88). The majority of the sample (51%) were on full care orders, with 38% on voluntary care orders, and 10% on other types of care orders. There were slightly more boys (52%) than girls (48%). The majority of the sample were White (76%), with 6% registered as Black, 10% mixed ethnicity and 8% another ethnicity (e.g. Middle Eastern). Six percent of the sample (*n* = 41) were registered as unaccompanied minors. Over their first three years in care, 73% spent the majority of their time in the care of a non-biological foster carer, 13% were with a kinship carer (e.g. grandparent), 11% were in residential care, 1% were in independent living, and less than 1% were with a biological parent. Demographics are broadly representative of national statistics on young people in care in England (e.g. National statistics: 64% enter care from school age (>5 years old); 56% boys; 72% in foster placement; 74% White, 8% Black and 10% mixed ethnicity; Department for Education, 2019).

**Table 1.** Sample Descriptives (N = 672).

Sex, % Male (*n*)	51 (342)
Ethnicity, % white (*n*)	76 (508)
Age entered care in years, *M* (*SD*), range	10.81 (4.08), 2–16yo
Number of placements^a^, *M* (*SD*), range	3.34 (2.57), 1–19
Missing person report^a^, % yes (*n*), range	32 (215), 0–109
Primary placement type^a,b^, % (*n*)	
Foster (non-biological) care	73 (484)
Kinship care	13 (87)
Residential care/semi-independent	14 (93)
Maltreatment history	
Neglect	71 (477)
Emotional abuse	67 (453)
Witnessed domestic violence	62 (415)
Physical abuse	49 (327)
Sexual abuse	19 (130)

*Note.* Sample excludes young people aged under 2-years old when they entered care, who were too young to have a valid SDQ completed.

^a^All over first 3 years in care system.

^b^*n* = 5 living with biological parent; *n* = 5 missing data.

As the primary aim of this study was to explore how SDQ scores changed, using the SDQ validated for 4–17 year olds, we could necessarily only include young people who would have been old enough to have a valid SDQ completed in either their first or second year in care. From the full sample, 70 children (9%) were aged 2 years old or younger when they first entered care. For these children they would have been too young within their first 2 years in care to have a valid carer SDQ completed. Consistent with this, 90% had no SDQ completed in these years. These 70 children were excluded from all analyses, leading to a total sample of 672 (sample characteristics presented in Table 1). Consistent with the initial full sample, 52% were boys, young people had moved in to care between 2.2 years old and 15 years old (*M* = 10.43, *SD* = 3.88), and 78% spent the majority of their first 3 years in care in the care of a non-biological foster carer. All further analyses refer to this final sample (*N* = 672), unless otherwise specified.

### Data Extraction and Measures

All data were extracted from electronic service records. In most cases, the SDQ was entered item-by-item either electronically or via a scanned paper copy of the original measure. Where available, SDQ data were extracted for each of the first 3 years that the young person was in care. Year 1 (Y1) was any carer-report SDQ completed within the first 12-months of entering care; Year 2 (Y2) was any completed between 13 and 24 months; and Year 3 (Y3) was any completed between 25 and 36 months. As is typical of service data, there were relatively large amounts of missing data (correlates of missingness discussed in Data Analytic Plan). There was no evidence that the year they entered care (i.e. 2012–2016) was significantly associated with SDQ missingness (*p* = .08). In Y1 38% (*n* = 257) had no SDQ, with 35% (*n* = 236) and 40% (*n* = 268) missing SDQs in Y2 and Y3, respectively. Overall, only 186 young people (28%) had an SDQ completed in each year, while 274 (41%) had two SDQs and 149 (22%) had one SDQ. Of the 672 young people, 63 (9%) did not have any SDQ recorded as completed at any point over their first 3 years in care.

#### Mental Health

Emotional and behavioural difficulties were measured via carer-report on the SDQ (Goodman, 2001), a widely used and validated measure of internalising and externalising problems in 4–17 year olds. Twenty-items (five per subscale) cover internalising (two subscales: emotional problems and peer problems) and externalising (two subscales: attention problems and conduct problems) difficulties, with five additional items measuring pro-social skills. Each item is rated on a 3-point Likert scale from 0 (*Not True*) to 2 (*Certainly True*), resulting in subscale score ranges of 0–10 and a total problem score range of 0–40. Consistent with original reporting of psychometrics for the SDQ and further reviews of the literature on its psychometric properties (Goodman, 2001; Stone et al., 2010), internal consistency for the problem subscales for this current sample were all adequate (α > .77; peer problems α = .67). To describe the overall mental health of the sample, we used the established 3-band SDQ categorisation system, where scores are divided in to ‘normal’, ‘borderline’ and ‘abnormal’ (freely available on the SDQ website).

#### Service Data Information

We also extracted basic descriptive information that may categorise a young person’s risk when they enter care, as well as markers of instability once they are in care. Descriptives included (i) age of removal (a broad proxy of length of maltreatment exposure), (ii) sex (0 = female, 1 = male) and (iii) maltreatment history. The latter information was gathered from chronologies, court reports, and police reports, coded as ‘present’ or ‘absent’ for sexual abuse, physical abuse, emotional abuse, witnessing domestic violence and neglect. Maltreatment history was collected to provide basic descriptive information, but was not included in further analyses as the extent of maltreatment experienced can vary substantially from information provided in initial reports. We also extracted data on ethnicity, which was ultimately coded as (0) White, (1) Any minority ethnicity, due to unequal group sizes. Indices of instability covered (i) total number of placement providers over their first 3 years in care, (ii) whether or not they were separated from their siblings: (0) separated from all siblings and (1) together with at least one sibling, and (iii) total number of missing person reports over their first 3 years in care. As most of the sample (68%) had no missing person reports recorded, this variable was coded categorically as (0) no missing person report recorded or (1) at least one missing person report over first 3 years. For placement provider, each placement provider was counted once regardless of how many times the young person had lived with them over the 3 years, or for how long.

### Data Analytic Plan

SDQ data were considered Missing at Random. An independent samples t-test showed those who had at least one SDQ completed (v none) were significantly younger when they entered care (*p* < .001; *M* = 10.63 years old, *SD* = 4.05 v *M* = 12.50, *SD* = 4.07). Having at least one SDQ completed (v none) was also associated with having slightly more placements (*p* = .01; *M* = 3.37, *SD =* 2.60 v *M* = 2.88, *SD* = 1.80), although total placements was not associated with the total number of SDQs completed (*p* = .81). Having at least one SDQ completed (v none) was not associated with the sex of the child (*p* = .59), or whether or not they had a missing person report (*p* = .46). Total problem scores from the previous year were also not associated with increased likelihood of the SDQ being completed the following year (*ps* > .13).

The primary question was how internalising (emotional and peer) and externalising (conduct and attention) difficulties develop after a young person enters care. As SDQ subscale scores were only moderately correlated (see supplementary table S1), we explored each four problem subscales individually. Initial analyses used growth mixture models (GMM) but entropy scores showed even for the best fitting models classification accuracy was only moderate, making them inappropriate for use as discrete classes in regression analyses (full GMM output are provided as supplementary material). We were also concerned that the classes generated via GMM masked substantial variation based on raw scores, bringing in to question their clinical utility (e.g. young people categorised as chronic, whose raw scores in fact showed recovery).^1^ Therefore, to provide a more in-depth exploration of how SDQ scores changed over time, we categorised participants a priori into one of four categories based on their repeatedly measured raw SDQ scores: (i) resilient [scores in normal range at all time points]; (ii) chronic [scores in borderline-abnormal range at all points]; (iii) delayed [scores in normal range at first SDQ, but borderline-abnormal range at later SDQ]; (iv) recovery [scores in borderline-abnormal range at first SDQ, but normal range at later SDQ]. This analysis could only include participants who had at least two SDQs completed (69% of total sample). There was no evidence of significant differences in the SDQ total problem scores, sex, ethnicity, age entered care or markers of instability for those who had one SDQ completed v those who had two or three completed (*ps* > .05). Their first SDQ was taken from their earliest available SDQ (i.e. either Y1 [*n* = 354] or Y2 [*n* = 106]).

Our secondary aim was to explore whether emotional and behavioural difficulties were associated with markers of instability in care. First, we used bivariate and point biserial correlations to understand basic associations between our markers of instability (number of placement providers, missing person reports, sibling-living arrangement) and total scores on our SDQ subscales. As a sensitivity check we also re-ran these analyses using multiple imputation for missing SDQ scores, using 50 iterations and predictive mean matching. There was no difference in the pattern of results, so supplementary materials present the associations using the raw data. Next, using our SDQ categories from Aim 1, we ran four separate multinomial logistic regressions with problem category as the outcome, for each of the SDQ problem subscales. These regressions explored associations between the three categories of instability, with sex, age entered care, and ethnicity added as covariates where appropriate. In each regression the resilient category was used as the reference category. As a final sensitivity check, as type of placement was associated with SDQ scores (described later), the main analyses were re-run only including those young people who were in a non-biological foster placement (i.e. the most common type of placement for children in care). Again, the pattern of results was largely the same. Discrepancies are noted in text.

## Results

### Descriptives and Preliminary Analyses

Associations between all variables and the proportion (and *n*) of the sample to fall into the ‘normal’, ‘borderline’ and ‘abnormal’ score range in each year are presented in supplementary Tables S1-S2. Consistent with national data of children in care in England (Department for Education, 2019), approximately 30–40% of the sample were rated in the abnormal range each year, with a further 9–17% in the borderline range. Based on paired-samples t-tests, there was no evidence of significant change in either total problem scores or any problem subscale scores between Y1 and Y2 (*ps* > .27) or Y1 and Y3 (*ps* > .44), with the sole exception of a small but statistically significant reduction in carer-rated emotional problems from Y1 to Y3 (reflecting a less than 0.5 mean change; *d* = .13, *p =* .046).

The primary type of placement they were in over their first 3-years in care (foster v kinship v residential) was not associated with total problem scores in Y1, but was in Y2 and Y3 (see supplementary Table S4). In Y2, there were significant differences between all three types of placements (*ps < .*02), with the highest total problem scores reported for those in residential care, then those is foster care, then those in kinship care. In Y3, there were no significant differences between total problem scores for those in foster versus kinship care (*p* = .79), but those in residential care had higher reported problems than those in either foster or kinship placements (*ps* < .001).

### Problem Symptom Categories

**Figure 1.**
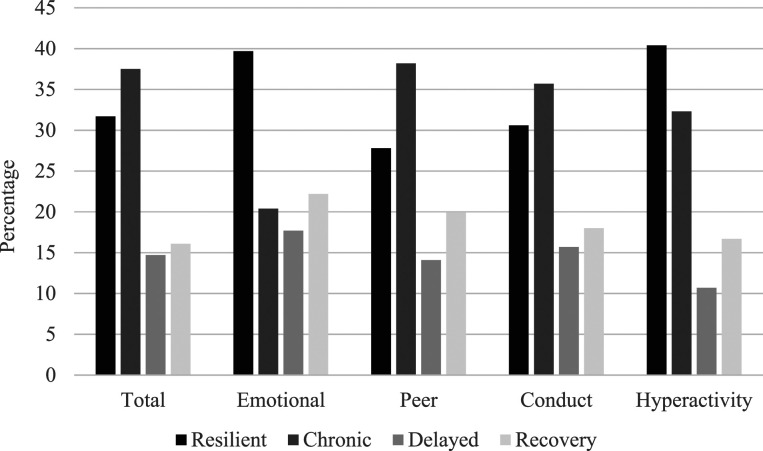
Percentage of Sample in Each Problem Category for Total SDQ Scores and Subscales.

The proportions of the sample in each of the four categories (resilient, chronic, delayed, and recovery) are presented in Figure 1. Ten cases (∼2% of sample) could not be classified in to one of the four categories, as their scores in each of the 3 years moved between the normal and abnormal range in no clear pattern. For all subscales, the most common groups were the resilient group and the chronic difficulties group. Delayed vulnerability and recovery profiles were less common (see Figure 1). Based on total problem scores from Y1 to Y2 (*n* = 276) and Y1 to Y3 (*n* = 237), as expected, paired samples t-tests confirmed no significant change in SDQ raw scores in the resilient group (*p* > .90) or the chronic group (*p* > .55), a significant increase in difficulties in the delayed vulnerability group (Y1-Y2, *d* = .99, *p* < .001; Y1-Y3, *d* = 1.82, *p* < .001), and decrease in the recovery group (Y1-Y2, *d* = .28, *p* = .05; Y1-Y3, *d* = 1.18, *p* < .001).

### Associations Between Descriptives, Markers of Instability and SDQ Scores

Bivariate and point-biserial associations between potential covariates (age, sex and ethnicity), markers of instability (total placements, missing person report, and sibling-living status) and the raw SDQ scores are in supplementary Table S3. Evidence of associations between the potential covariates and SDQ scores were mixed and inconsistent. Entering care at a younger age was associated with higher hyperactivity scores in all years and greater internalising (peer and emotional) problems in Y2. To be consistent, age entered care was included as a covariate in all regressions. Sex was associated with hyperactivity in Y2 and Y3 and conduct problems in Y1 only, with higher mean scores for boys. As there was no evidence that sex of the child was associated with total problem scores or any individual categories across all years (*p* > .07) sex was not included as a covariate in later analyses. Ethnicity was significantly associated with total difficulties scores in each year, as well as with peer problems and hyperactivity, but not emotional difficulties or conduct. Where there were differences in each case, higher difficulties were associated with increased likelihood of being White. Ethnicity was included as a covariate in later analyses.

A higher number of placement providers and presence of a missing person report were both associated with greater internalising and externalising problems, particularly in Y2 and Y3, while being separated from all siblings was associated with greater internalising problems in all years (see Table 2 and supplementary Table S3).

**Table 2.** Descriptive Statistics for Age of Removal and Markers of Instability for Each SDQ Group.

	Age of Entering Care in years, *M* (SD), Range	Placements, *M* (SD), Range	Confirmed Missing Person Report, % (*n*)	Living Separate from all Siblings, % (*n*)
Emotional Problems				
Resilient	9.99 (4.02), 2.25–15.92	3.08 (2.06), 1–10	24%, 43	56%, 78
Chronic	11.19 (3.75), 2.17–15.92	4.11 (3.10), 1–15	42%, 39	78%, 58
Delayed	10.32 (4.01), 2.50–15.92	3.23 (2.71), 1–18	33%, 26	67%, 42
Recovery	10.73 (3.60), 2.50–15.92	3.18 (2.17), 1–11	27%, 27	68%, 47
Peer Problems				
Resilient	10.01 (4.08), 2.17–15.92	2.71 (1.89), 1–11	20%, 25	46%, 43
Chronic	10.89 (3.63), 2.58–15.92	3.73 (2.91), 1–18	34%, 59	78%, 115
Delayed	9.77 (4.05), 2.50–15.83	3.28 (2.05), 1–8	38%, 24	59%, 31
Recovery	10.61 (3.93), 2.83–15.88	3.50 (2.39), 1–14	29%, 26	39%, 38
Conduct Problems				
Resilient	10.95 (4.00), 2.17–15.92	2.52 (1.91), 1–11	16%, 22	62%, 60
Chronic	10.10 (3.82), 2.50–15.92	4.09 (2.92), 1–18	40%, 64	71%, 93
Delayed	9.82 (3.90), 3.08–15.92	3.12 (2.12), 1–9	28%, 20	60%, 34
Recovery	10.71 (3.70), 2.50–15.92	3.58 (2.32), 1–14	30%, 24	64%, 38
Hyperactivity				
Resilient	11.29 (3.91), 2.17–15.92	2.96 (2.31), 1–18	24%, 44	60%, 81
Chronic	9.98 (3.63), 2.58–15.92	3.61 (2.75), 1–14	35%, 51	73%, 86
Delayed	10.19 (4.24), 2.25–15.67	4.04 (3.00), 1–15	33%, 16	69%, 27
Recovery	9.74 (3.73), 2.50, 15.58	3.43 (1.94), 1–8	29%, 22	63%, 32

#### Multinomial Regressions for SDQ Groups

Table 2 provides descriptive statistics for age entered care and the three markers of instability (sibling status, missing person report and placements) for each SDQ score category. Results of multinomial regression models in which these variables are entered simultaneously as predictors of SDQ groupings are presented in Tables 3 and 4.

**Table 3.** Results of Multinomial Logistic Regressions for Internalising Problems Subscales.

	Emotional Problems					Peer Problems			
	*N*	B(SE)	Wald (1)	Exp(B), 95%CI		*n*	B(SE)	Wald (1)	Exp(B), 95%CI
Chronic	74					147			
Age, years^a^		0.002 (0.05)	0.001	1.00 (0.91, 1.10)			0.07 (0.05)	2.47	1.07 (0.98, 1.17)
Ethnicity^a^		0.12 (0.37)	0.11	1.13 (0.54, 2.35)			0.90 (0.36)^**^	6.39	2.46 (1.22, 4.93)
Placements^a^		0.16 (0.06)^**^	6.52	1.17 (1.04, 1.32)			0.22 (0.07)^**^	8.98	1.25 (1.08, 1.44)
Missing person^a^		−0.02 (0.40)	0.004	0.97 (0.45, 2.14)			0.45 (0.41)	1.24	1.57 (0.71, 3.50)
Sibling-status^a^		0.91 (0.36)^*b^	6.29	2.47 (1.22, 5.01)			1.28 (0.32)^**^	15.83	3.60 (1.91, 6.75)
Delayed	63					52			
Age, years^a^		−0.008 (0.05)	0.03	1.04 (0.91, 1.12)			−0.06 (0.06)	1.04	0.94 (0.84, 1.06)
Ethnicity^a^		0.65 (0.43)	2.25	1.91 (0.82, 4.46)			0.81 (0.45)	3.21	2.26 (0.93, 5.60)
Placements^a^		0.04 (0.07)	0.37	1.04 (0.91, 1.20)			0.14 (0.09)	2.52	1.15 (0.97, 1.37)
Missing person^a^		0.02 (0.43)	0.003	1.02 (0.44, 2.36)			−0.75 (0.52)	2.09	0.47 (0.17, 1.31)
Sibling-status^a^		0.44 (0.35)	1.57	1.55 (0.78, 3.07)			0.41 (0.39)	1.11	1.52 (0.70, 3.28)
Recovery	69					55			
Age, years^a^		0.05 (0.05)	1.30	1.06 (0.96, 1.16)			0.05 (0.06)	0.89	1.06 (0.94, 1.18)
Ethnicity^a^		0.60 (0.42)	2.06	1.82 (0.81, 4.10)			0.98 (0.48)^*^	4.27	2.68 (1.05, 6.81)
Placements^a^		0.04 (0.07)	0.26	1.04 (0.90, 1.19)			0.19 (0.09)^*^	5.25	1.21 (1.03, 1.43)
Missing person^a^		0.30 (0.41)	0.55	1.35 (0.61, 3.02)			−0.09 (0.49)	0.04	0.91 (0.35, 2.39)
Sibling-status^a^		0.40 (0.34)	1.38	1.49 (0.77, 2.92)			0.70 (0.40)	3.05	2.00 (0.92, 4.40)

*Note.* In all cases the reference category is the resilient group. For emotional problems the resilient group comprised 139 subjects; for peer problems the resilient group comprised 93 subjects. *p < .05, ** *p* ≤ .01.

^a^Age is aged removed from care (in years); Ethnicity is coded as (0) White, (1) Minority ethnicity; Sex is coded as (0) female, (1) male; Placements is total number of placement providers over first 3-years; Missing person is whether there was no recorded missing person report over first 3-years (0) or at least one missing person report (1); Sibling-status is whether they were separated from all siblings (0) or lived with at least one sibling (1).

^b^For the sensitivity analysis of those in foster care only, this difference was non-significant (*B* = 0.78, *SE* = 0.40, *p* = .053).

**Table 4.** Results of Multinomial Logistic Regressions for Externalising Problems Subscales.

	Conduct Problems				Hyperactivity			
	*n*	B(SE)	Wald (1)	Exp(B), 95%CI	*n*	B(SE)	Wald (1)	Exp(B), 95%CI
Chronic	131				118			
Age, years^a^		−0.14 (0.05)^**^	9.39	0.87 (0.79, 0.95)		−0.17 (0.04)^**^	15.49	0.84 (0.77, 0.92)
Ethnicity^a^		0.06 (0.36)	0.03	1.07 (0.52, 2.17)		1.11 (0.38)^**^	8.56	3.04 (1.44, 6.39)
Placements^a^		0.32 (0.08)^**^	16.70	1.38 (1.18, 1.60)		0.13 (0.06)^* +^	4.55	1.14 (1.01, 1.28)
Missing person^a^		−1.08 (0.41)^**^	6.90	0.34 (0.15, 0.76)		−0.38 (0.36)	1.11	0.68 (0.34, 1.39)
Sibling-status^a^		0.43 (0.33)	1.72	1.54 (0.81, 2.92)		0.93 (0.32)^**^	8.65	2.53 (1.36, 4.70)
Delayed	57				39			
Age, years^a^		−0.07 (0.05)	1.71	0.93 (0.84, 1.04)		−0.13 (0.06)^*^	4.50	0.88 (0.78, 0.99)
Ethnicity^a^		0.08 (0.43)	0.04	1.09 (0.47, 2.50)		0.42 (0.47)	0.77	1.52 (0.60, 3.83)
Placements^a^		0.12 (0.10)	1.59	1.13 (0.94, 1.36)		0.17 (0.08)^*+^	4.93	1.18 (1.02, 1.37)
Missing person^a^		−0.68 (0.49)	1.97	0.51 (0.20, 1.31)		−0.04 (0.52)	0.01	0.96 (0.35, 2.64)
Sibling-status^a^		−0.04 (0.38)	0.01	0.96 (0.46, 2.01)		0.69 (0.44)	2.51	1.99 (0.85, 4.68)
Recovery	59				51			
Age, years^a^		−0.04 (0.06)	0.46	0.96 (0.86, 1.07)		−0.18 (0.06)^**^	10.06	0.84 (0.75, 0.93)
Ethnicity^a^		0.97 (0.54)	3.23	2.63 (0.92, 7.56)		0.63 (0.45)	2.01	1.88 (0.79, 4.51)
Placements^a^		0.24 (0.09)^**^	7.23	1.27 (1.07, 1.50)		0.13 (0.07)	3.08	1.14 (0.99, 1.31)
Missing person^a^		−0.74 (0.47)^+^	2.47	0.47 (0.19, 1.20)		−0.63 (0.47)	1.77	0.54 (0.21, 1.35)
Sibling-status^a^		−0.04 (0.39)	0.01	0.96 (0.45, 2.04)		0.40 (0.39)	1.07	1.49 (0.70, 3.19)

*Note.* In all cases the reference category is the resilient group. For chronic problems the resilient group comprised 97 subjects; for hyperactivity the resilient group comprised 135 subjects. *p < .05, **p <.01.

^+^ From the sensitive analysis of those in foster care only, for conduct problems, having a missing person report was significantly associated with increased likelihood of being in the recovery v resilient category (*B* = −1.18, *SE* = 0.54, *p* = .03); For hyperactivity having more placements no longer significantly differentiated those in the resilient v chronic group [although the effect sizes were similar; *B* = .13, *SE* = .07, *p* = .07] or the resilient v delayed group [although the effect sizes were similar; *B =* 0.15, *SE* = 0.10, *p* = .13].

^a^Age is aged removed from care (in years); Ethnicity coded as (0) White, (1) Minority ethnicity; Placements is total number of placement providers over first 3-years; Missing person is whether there was no recorded missing person report over first 3-years (0) or at least one missing person’s report (1); Sibling-status is whether they were separated from all siblings (0) or lived with at least one sibling (1).

#### Emotional Problems

Having more placement providers and living separately (v together) from siblings were both uniquely associated with greater likelihood of being in the chronic problems group v resilient group. There were no significant differences between those in the resilient group and either the recovery or delayed vulnerability groups.

#### Peer Problems

Being White (v Minority ethnicity), having more placement providers and being separated from siblings (v together) were also associated with increased likelihood of being in the chronic v resilient group. More placements were also associated with increased likelihood of being in the recovery group (where peer problems were initially high) v resilient group.

#### Conduct Problems

Entering care at a later age, having more placement providers, and having at least one missing person report (v no report) were all associated with increased likelihood of being in the chronic problems v resilient group, while having more placements was also associated with increased likelihood of being in the recovery group (where conduct problems were initially high) v resilient group.

#### Hyperactivity

Moving into care earlier, being White, having more placements, and living separated from siblings, were all associated with increased likelihood of being in the chronic group v resilient group. Moving into care later was associated with increased likelihood of being in the delayed vulnerability group v resilient group, and the recovery v resilient group.

### Sensitivity Check for those in Foster Placement Only

Given there was some, albeit inconsistent, evidence that the primary type of placement the young person spent their first 3-years in was associated with SDQ scores, we re-ran the main analyses only for those young people in a non-biological foster placement (i.e. the most common type of placement). Multinomial regression analyses showed the pattern of results were largely the same as was found for the total sample (see above for main results). Effect sizes were similar, although a small number of findings were no longer statistically significant in this reduced sample (see footnotes of Tables 3 and 4).

## Discussion

We used routinely collected social care data to understand the development of both internalising and externalising problems over the first 3 years of being in the English out-of-home care system. Findings confirmed existing evidence of high rates of internalising and externalising problems in this group (Ford et al., 2007) and were in line with national data of cross-sectional rates of problems, based on the routinely collected carer-report SDQ (Department for Education, 2019). Prospective results showed that stable profiles were most common over the first 3 years of being in care, whether they were resilient and stable or reflecting chronically-elevated problems across all 3 years.

The chronicity of mental health difficulties was particularly evident for those SDQ subscales where difficulties were likely to be more easily observable to carers: peer problems, conduct problems and hyperactivity. Given the wide age range of young people in this study, besides being more easily observable to carers, the chronicity of these particular subscales may also reflect that age of onset of impulse-control related problems (e.g. hyperactivity) is generally found to be earlier than internalising/emotional difficulties, such as generalised anxiety (Kessler et al., 2007). On these subscales there was no evidence of mean change in symptoms from the first to second year of a young person being in care, or from the first to third year. The majority of young people were on persistent trajectories that were either resilient or chronically-elevated. From their first carer-report SDQ, approximately 50–60% of young people were rated as having elevated difficulties on these three domains (peer, conduct and hyperactivity). From this group, for approximately 70% of young people these problems were shown to persist based on later SDQ scores, with only around 30% having scores that moved to a ‘normal’ range (i.e. ‘recovery’). Understanding what factors may be promoting recovery remains a crucial area of research. Of course, recovery or resilience to early maltreatment is a complex area, likely encompassing individual and system-level factors (McCrory & Viding, 2015). Identifying mechanisms that may promote recovery from initially elevated symptoms v more chronic problems, including at different ages or developmental stages, remains a crucial area of work, particularly to guide service-providers in their knowledge of which young people may require more timely intervention.

Expectedly, the chronicity of these problems was related to the stability of the young person’s care experience. In particular, greater internalising and externalising problems were both associated with the number of placement providers over the first 3 years in care. We also found basic associations between the primary type of placement the young person was in over their first 3-years and total difficulty scores, with those is residential care having greater difficulties. Caution is warranted in drawing conclusions on causation here, as young people in residential care are likely to be older/teenagers, when we would expect a rise in mental health difficulties, while in many cases they would have had numerous failed foster placements prior to being placed in residential care. Overall, findings support previous, largely US-based studies, that have highlighted links between increased mental health difficulties and placement (in)stability (Newton et al., 2000), as well as the larger body of qualitative work highlighting the importance of having a single trusted adult to turn to for support, in promoting the wellbeing of young people in care (Ahrens et al., 2011; Selwyn et al., 2017). Associations between mental health and placement stability is a likely cyclical relationship, with young people with greater behaviour or emotional difficulties potentially more difficult for carers to manage (e.g. increasing carer stress, which is then related to poorer child mental health; Goemans et al., 2020), but the breakdown of placements also leading to further entrenchment of the young person’s difficulties (e.g. Gilbertson & Barber, 2003; Rock et al., 2013; Sinclair & Wilson, 2003). Despite consistent evidence of the importance of placement stability, there remains limited high quality empirical evidence (particularly longitudinal) to understand foster carer characteristics that might support or hinder either placement stability or child mental health (Orme & Buehler, 2001). Such evidence is important for supporting matching between children and carers, and for developing carer training and support packages focused on maintaining stability. Although interventions that target processes such as reducing stress or increasing sensitive parenting may be useful, the empirical evidence for many carer-focused interventions remains limited and there remains limited focus on identifying the mechanisms that might drive improvements in young people (Schoemaker et al., 2020). It is also worth noting that our research here shows that many young people are experiencing high emotional and/or behavioural difficulties from their first year in care. These young people are likely to require professional mental health support, but chronic underfunding means such support is often not available even with significant advocacy from carers (Hiller et al., 2020).

Being separated from all siblings was also associated with greater internalising and externalising problems, and particularly with chronically elevated internalising problems, while having a missing person report was more specifically relevant to young people with elevated conduct problems. It remains a priority of most children’s services to place siblings together, but practicalities can also make this challenging (e.g. finding a placement provider who can take multiple siblings). Nevertheless, this work adds to the growing body of international literature of the potential negative consequences of placing young people without any siblings (Hegar, 2005; Jones, 2016), with sibling separation potentially further eroding a young person’s sense of belonging (Leather, 2005). Although our work further supports the negative consequences of sibling separation, it is also important to acknowledge the challenges of decision-making around sibling placements. Some research has shown that young people separated from their siblings can do just as well as those placed together (Jones, 2016), while there can be genuine safety reasons that a young person may need to be placed separately. Nevertheless, based on our research and the wider literature, seperating siblings should clearly only occur when absolutely neccessary for the welfare of the child. If it is occuring for other reasons (e.g., resource-driven) this should be urgently addressed. Like placement instability, associations between separation from siblings and mental health difficulties is likely to be reciprocal, with separation having the potential to cause elevated distress, but also where more complex mental health difficulties may lead practitioners to decide a singleton placement could aid better support or recovery (whether that may be true or not). Unpacking these potential bidirectional associations remains another important area for future research.

### Clinical Implications

Primarily, this work highlights the need for more timely and targeted mental health support for young people in care. Moving into care, where physical safety is prioritised, is not enough of an intervention to expect improved mental health outcomes. While government-reported cross-sectional data have consistently shown elevated rates of internalising and externalising difficulties in this group, the current longitudinal work highlights that over time, these difficulties largely commonly remain chronic and pervasive. That is, based on carer report, for most young people who have elevated difficulties in their first year, these problems will persist rather than naturally recover. Similarly, findings suggest waiting for placement stability before providing mental health support for young people in care is likely to mean those with the greatest needs will only find their difficulties (and thus instability) worsening. Why many young people in care experience persistently elevated internalising and/or externalising difficulties, despite decades of efforts to address them mental health of this group, remains a crucial area of research. In the UK, like many other developed countries, there are significant ongoing capacity issues in children’s social care and child and adolescent mental health services. However, there have also been concerns expressed about the under-identification of common mental health problems in this group, which can have an impact on evidence-based treatment decision-making (e.g. Woolgar & Baldock, 2015). Any efforts to effectively target the mental health needs of this group are likely to require a coordinated effort between social care and (mental) health care settings, with a dual focus on broader emotional wellbeing and on the evidence-based targeting of diagnosable mental health disorders.

### Strengths and Limitations

This study has many strengths, including the relatively large sample size across multiple local authorities and the novel evidence for the prospective development of both internalising and externalising problems in this vulnerable and under-researched group. It also provides an example of what can be done with existing but under-utilised social-care service data. However, findings should also be considered in light of limitations, which primarily stem from the reliance on service data. First, there were large amounts of missing data. While missingness was not associated with key variables, including initial SDQ severity scores, it may have been associated with other factors that were not measured here. Of note, basic associations were robust to the use of multiple imputation. Second, there is potential for measurement error. The extrapolation of data from the service files was quality checked, but the accuracy of the available data depends on the site accurately inputting that information into the file. This limitation is inherent in any work with service data. However, measurement error may have inevitably impacted our groupings, which relied on raw data. Of note, when using GMM analyses the pattern of results was similar, as were correlational findings. Relatedly, we also used the three-band SDQ score classification system. This was because this system has been the most thoroughly researched. However, recent preliminary evidence suggests young people in care may require lower thresholds, in which case our findings may be an underestimation of rates of difficulties (Wright et al., 2019). Next, because the SDQ used is validated for 4–17 year olds, we also necessarily excluded children who entered care under 2 years old age. Thus, findings may not be generalisable to very young children. Third, SDQs are based on carer report and in many cases, carers changed from 1 year to the next. The available data meant we were unable to whether this may have impacted results, including whether length of time in a placement was associated with SDQ completion or scores. This may particularly affect reporting on the emotional problems subscale, where items reflect more internal states. That said, a large study of young people in care, using multi-informant SDQs, found that carer report SDQs were robust predictors of independently assessed psychopathology (Goodman et al., 2004). Fourth, our analyses were unable to account for the potential role of neurodevelopmental disorders (NDDs), but it is important to note that rates of NDDs are elevated in this group (Ford et al., 2007). The complex associations between maltreatment, neurodevelopmental disorders, and broader mental health remains a crucial area of research. Finally, as we were exploring longitudinal profiles, the study only included young people who had been in care for at least two and a half years, so findings cannot necessarily be generalised to those who enter care for a shorter period of time. Similarly, if young people moved local authorities within that period, we would also likely not have captured them here. It is also the case that the local authorities were selected based on opportunity and because their service files were electronic. Although there may be some variation in profiles between different local authorities, our sample demographics were broadly in line with national demographics of young people in care, while our cross-sectional rates of elevated SDQ scores were also consistent with national data. These limitations notwithstanding, this work addresses an important gap in our empirical knowledge of the mental health of this group of young people (Tarren-Sweeney & Goemans, 2019).

## Summary

In sum, this work provides important insight in to the development of internalising and externalising problems when young people enter the out-of-home care system. Based on carer report, while many young people were relatively resilient to their early experiences and showed low levels of problems, approximately half of the sample were on more problematic, often chronic, trajectories. Where young people were experiencing problems in their first year in care, recovery profiles were less common, and chronic problems were relatively robustly associated instability in care, thereby incurring both personal and economic consequences. Findings highlight the importance of policy and practice across social care and mental health contexts in providing these young people with more timely and intensive evidence-based early interventions, to promote recovery from internalising and/or externalising problems and avoid continued cycles between elevated mental health difficulties and ongoing instability in care.

## Supplemental Material

sj-pdf-1-cmx-10.1177_10775595211070765 – Supplemental Material for The Development of Young Peoples’ Internalising and Externalising Difficulties Over the First Three-Years in the Public Care System Click here for additional data file. Supplemental Material, sj-pdf-1-cmx-10.1177_10775595211070765 for The Development of Young Peoples’ Internalising and Externalising Difficulties Over the First Three-Years in the Public Care System by Rachel M Hiller, Abigail Fraser, Megan Denne, Andreas Bauer and Sarah L Halligan in Child Maltreatment

## Notes

1. Our initial plan was to use growth mixture modelling (GMM) in MPlus to identify clusters of individuals with similar patterns, across the four problem subscales, with age entered care entered as a covariate in all analyses. We modelled linear, quadratic and intercept models, using Bayesian Information Criterion (BIC) and Akaike Information Criterion (AIC) to judge model fit. Entropy and classification probability scores were used to gauge classification reliability, with values of 0.40, 0.60 and 0.80, representing low, medium, and high class separation, respectively (Clark & Muthén, 2009). However, the best fit models (in all cases, 3-class linear models) showed only medium entropy values (i.e. class assignment could not be made with confidence). Figures for the GMM analyses are in the supplementary materials.
