# Supplementary material for: The Development of Young Peoples’ Internalising and Externalising Difficulties Over the First Three-Years in the Public Care System
Source: Child Maltreat. 2022 Feb 1;28(1):141–51. doi: 10.1177/10775595211070765 (PMC9716486; doi:10.1177/10775595211070765)
Supplement: sj-pdf-1-cmx-10.1177_10775595211070765 – Supplemental Material for The Development of Young Peoples’ Internalising and Externalising Difficulties Over the First Three-Years in the Public Care System [file sj-pdf-1-cmx-10.1177_10775595211070765.pdf]

Supplementary Material

GMM output for 3-class linear models

Emotional Problems (Entropy = .65)

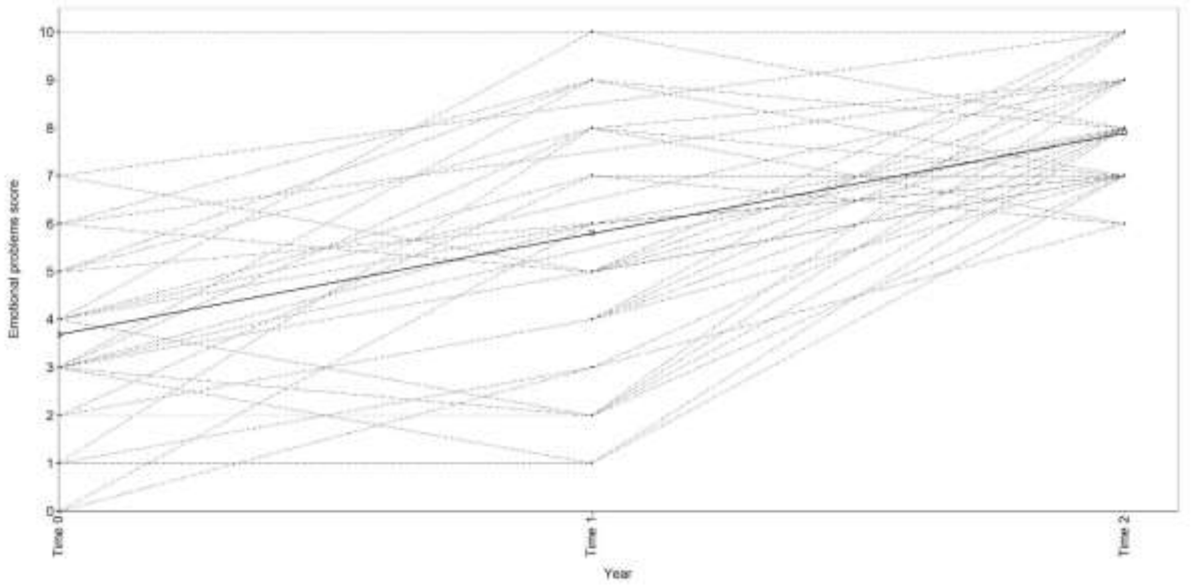

Class 1: Chronically worsening (11.0%)

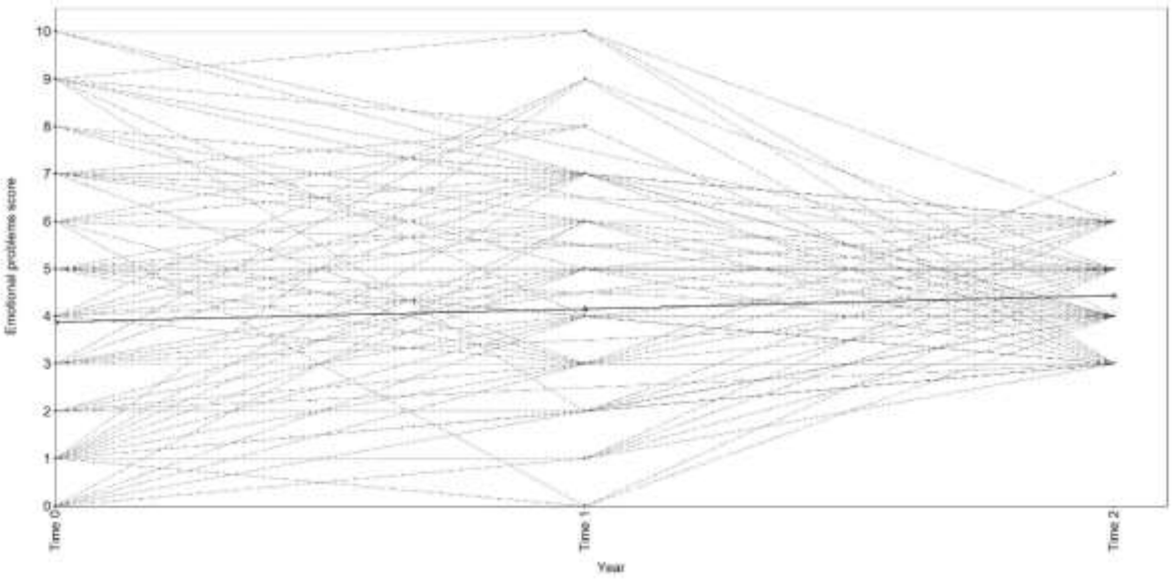

Class 2: Chronic stable (32.8%)

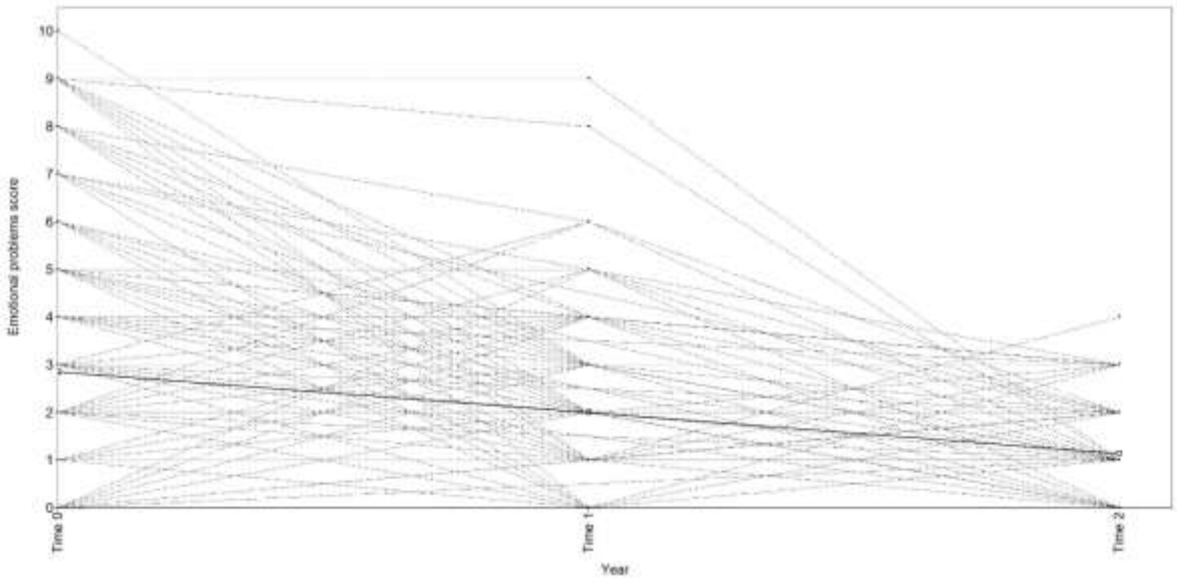

Class 3: Relatively resilient (56.2%)

Mental health of young people in care

Conduct Problems Classes (Entropy = .65)

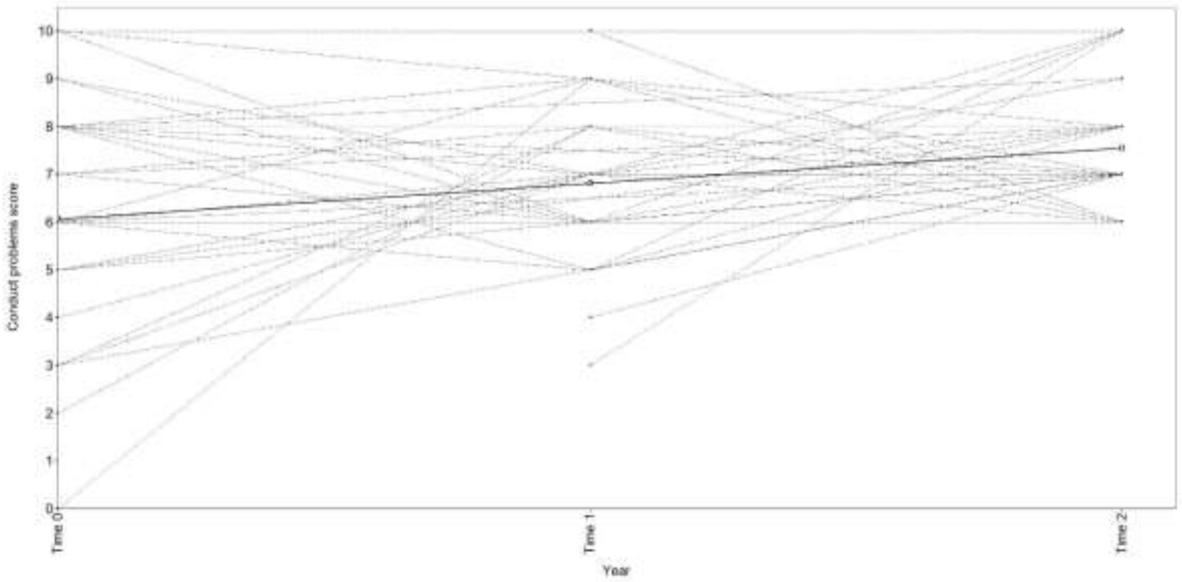

Class 1: Chronically worsening (11.6%)

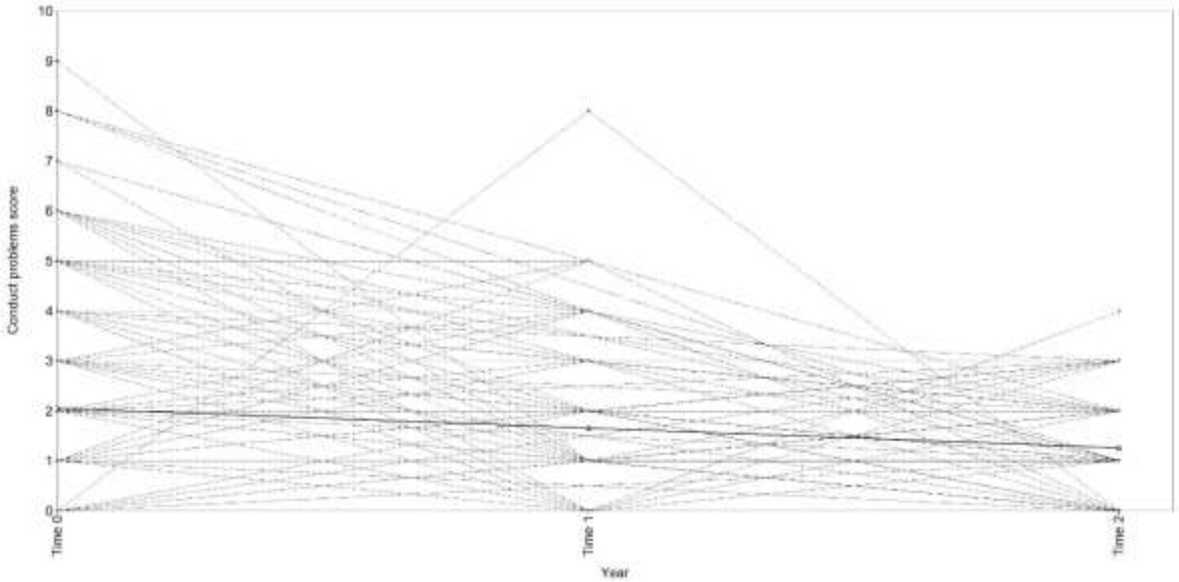

Class 2: Resilient (56.4%)

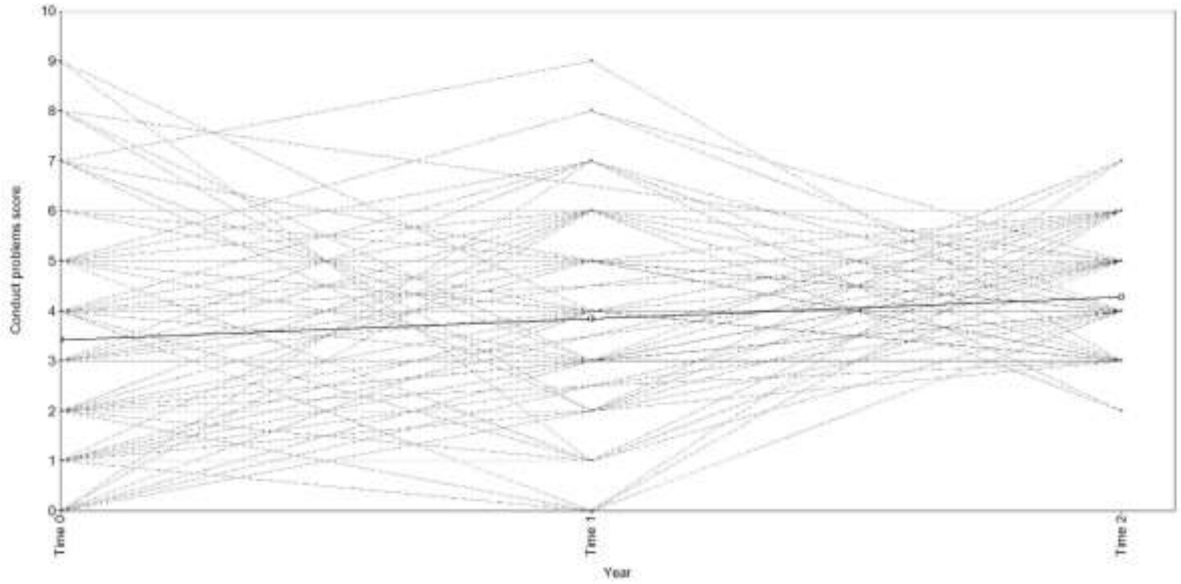

Class 3: Borderline-Chronic (31.8%)

Peer Problems Classes (Entropy = .67)

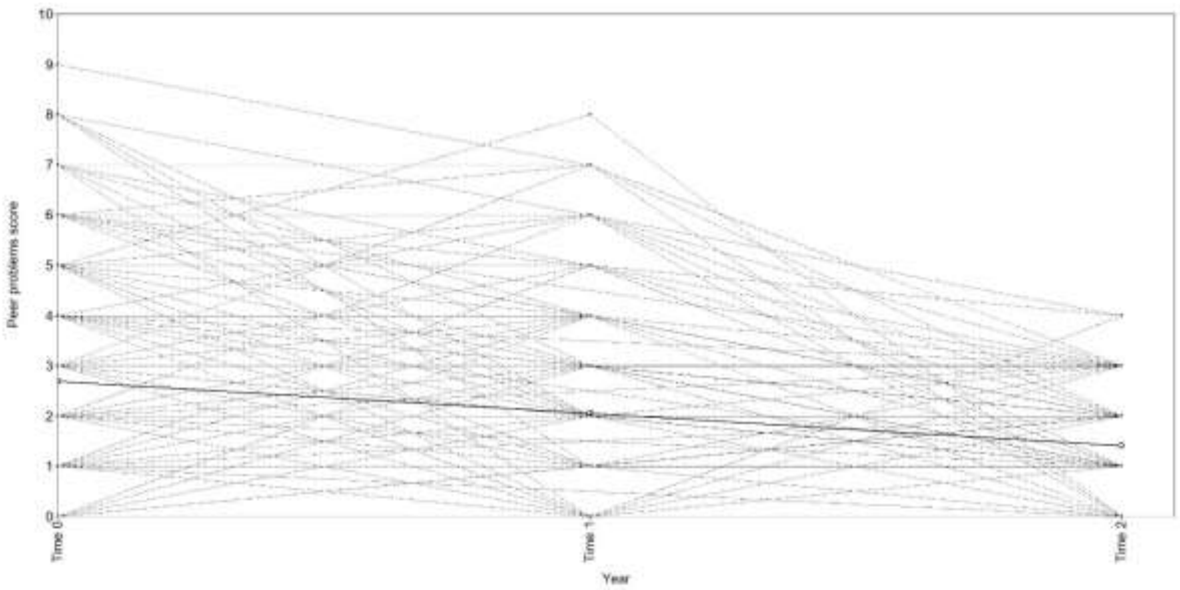

Class 1: Resilient (66.2%)

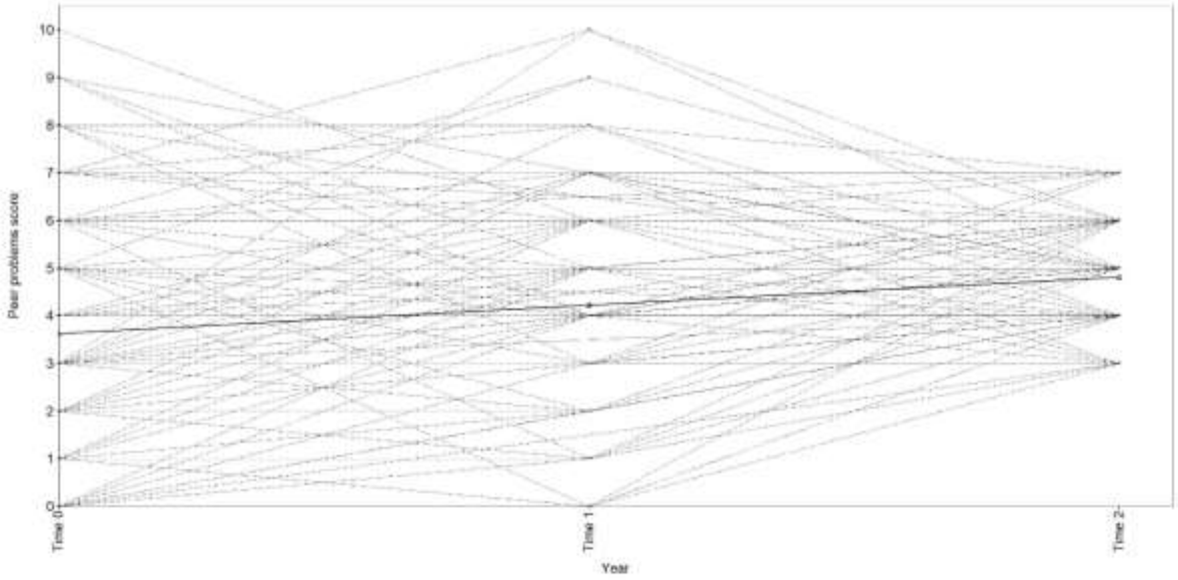

Class 2: Chronic stable (29.6%)

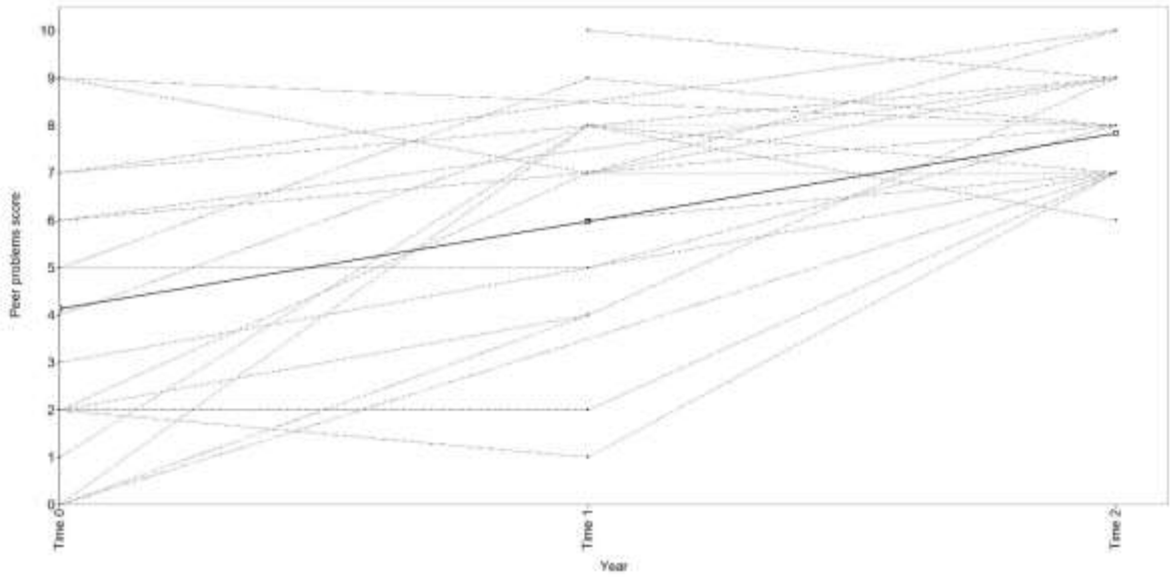

Class 3: Chronically worsening (4.2%)

Mental health of young people in care

Hyperactivity Classes (Entropy = .59)

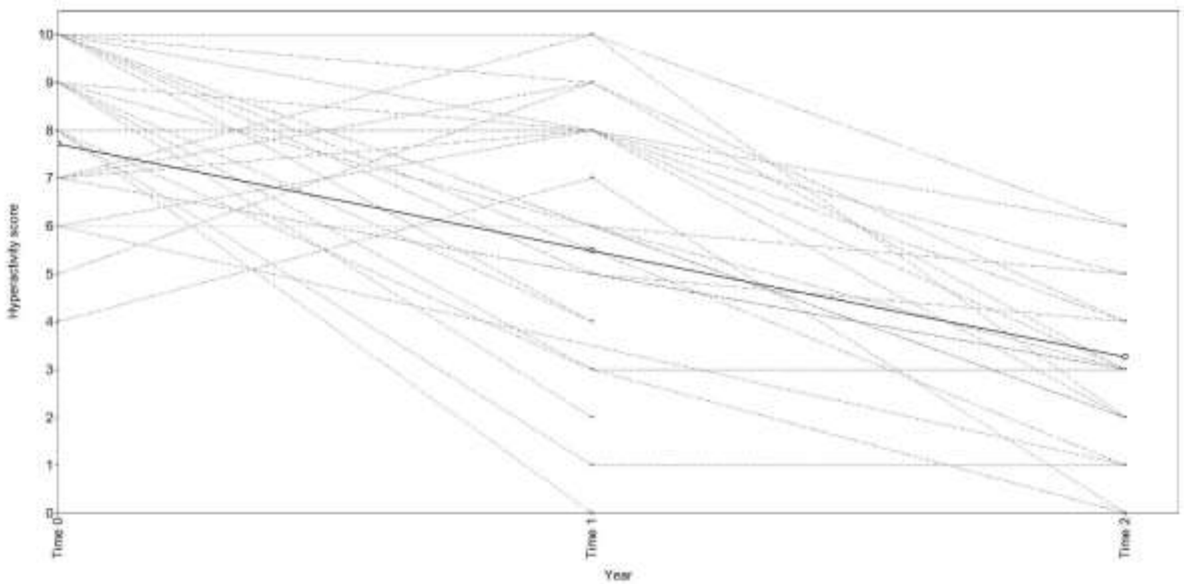

Class 1: Recovery (5.9%)

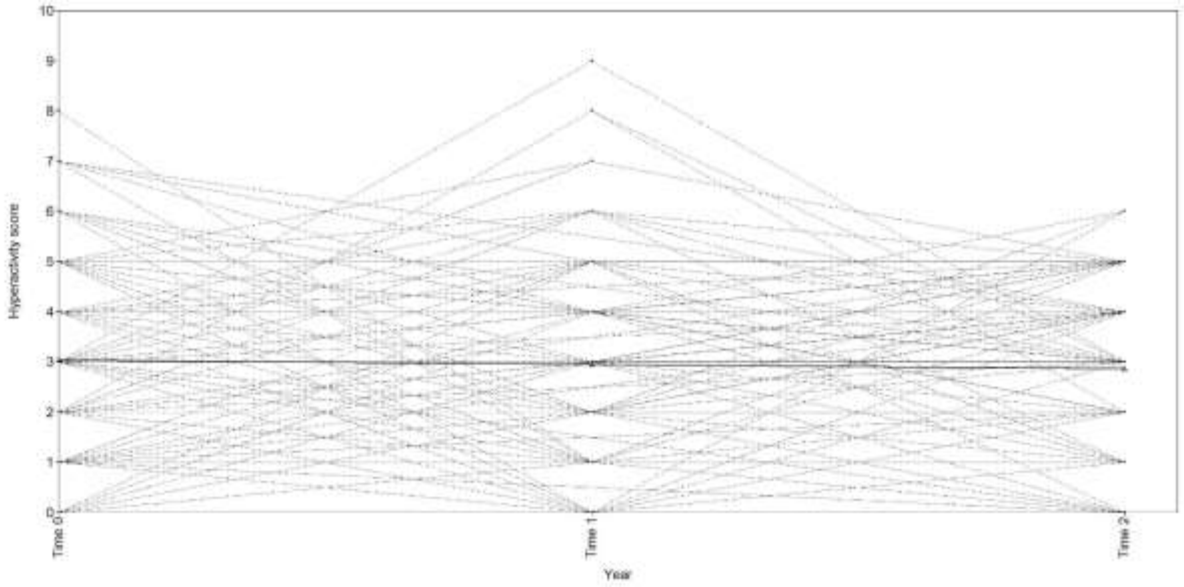

Class 2: Resilient (49.8%)

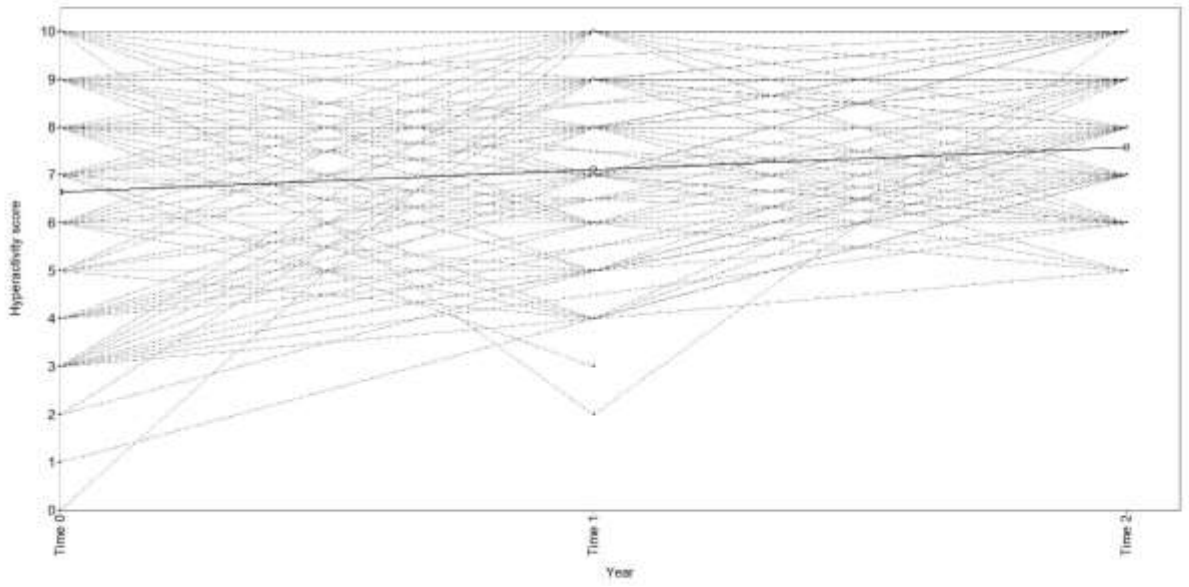

Class 3: Chronic (44.2%)

Table S1. *Associations between SDQ total score and subscales at each time point*

	1.	2.	3.	4.	5.	6.	7.	8.	9.	10.	11.	12.	13.	14.	15.	16.	17.
1. Y1 TOT																	
2. Y1 EMOT	.69**																
3. Y1 PEER	.76**	.45**															
4. Y1 COND	.76**	.28**	.46**														
5. Y1 HYPER	.80**	.34**	.43**	.56**													
6. Y1 PRSC	-.48**	-.15**	-.44**	-.50**	-.36**												
7. Y2 TOT	.58**	.32**	.47**	.47**	.48**	-.37**											
8. Y2 EMOT	.37**	.42**	.27**	.20**	.21**	-.17**	.67**										
9. Y2 PEER	.41**	.20**	.47**	.30**	.28**	-.31**	.75**	.41**									
10. Y2 COND	.50**	.20**	.38**	.53**	.40**	-.30**	.76**	.31**	.44**								
11. Y2 HYP	.48**	.14*	.33**	.38**	.56**	-.34**	.78**	.30**	.42**	.53**							
12 Y2 PRSC	-.35**	-.17**	-.31**	-.29**	-.27**	.42**	-.47**	-.20**	-.41**	-.46**	-.36**						
13. Y3 TOT	.51**	.23**	.34**	.46**	.47**	-.27**	.69**	.40**	.51**	.56**	.62**	-.40**					
14. Y3 EMOT	.28**	.26**	.19**	.21**	.17**	-.05	.40**	.51**	.27**	.24**	.23**	-.21**	.70**				
15. Y3 PEER	.50**	.30**	.43**	.38**	.35**	-.19**	.55**	.24**	.60**	.38**	.45**	-.37**	.77**	.42**			
16. Y3 COND	.41**	.11	.25**	.50**	.37**	-.32**	.51**	.20**	.33**	.59**	.44**	-.34**	.78**	.34**	.50**		
17. Y3 HYP	.40**	.07	.20**	.35**	.54**	-.27**	.60**	.26**	.35**	.47**	.71**	-.29**	.82**	.39**	.48**	.57**	
18. Y3 PRSC	-.26**	-.15*	-.24**	-.27**	-.13*	.40**	-.38**	-.14*	-.32**	-.39**	-.33**	.50**	-.45**	-.17**	-.44**	-.49**	-.29**

Note. TOT = total problem score; EMOT = total score on emotional problems subscale; PEER = total score on peer problems subscale; COND = total score on conduct problems subscale; HYP = total score on hyperactivity subscale; PRSC = total score on prosocial subscale. In all cases, higher scores mean higher endorsement of subscale (in all cases more significant difficulties, except for prosocial scale).

*$p < .05$, **$p < .01$

Table S2. *Proportion of sample in normal, borderline, and abnormal problems range at each time point, alongside means and standard deviations.*

	Normal % (*n*)	Borderline % (*n*)	Abnormal % (*n*)	*M* (*SD*)
		**Year 1**		
Total	50 (204)	13 (54)	37 (149)	14.40 (7.92)
Emotional	56 (232)	10 (41)	34 (140)	3.37 (2.74)
Peer	44 (181)	17 (71)	39 (158)	3.08 (2.29)
Conduct	50 (207)	12 (48)	38 (158)	2.97 (2.56)
Hyperactivity	56 (233)	11 (47)	32 (134)	5.01 (2.97)
Prosocial	60 (246)	15 (61)	25 (102)	6.33 (2.63)
		**Year 2**		
Total	47 (206)	12 (51)	41 (180)	14.50 (7.74)
Emotional	63 (275)	9 (39)	29 (125)	3.09 (2.54)
Peer	46 (202)	11 (50)	42 (185)	3.14 (2.47)
Conduct	45 (198)	16 (71)	39 (169)	3.15 (2.45)
Hyperactivity	55 (240)	11 (50)	34 (149)	5.15 (2.95)
Prosocial	66 (291)	14 (60)	20 (88)	6.49 (2.63)
		**Year 3**		
Total	47 (190)	13 (53)	40 (164)	14.32 (7.97)
Emotional	61 (248)	13 (51)	27 (109)	3.04 (2.57)
Peer	49 (200)	14 (55)	38 (153)	3.01 (2.38)
Conduct	48 (195)	13 (51)	40 (162)	3.10 (2.49)
Hyperactivity	55 (222)	10 (42)	35 (143)	5.16 (2.94)
Prosocial	70 (284)	12 (47)	19 (77)	6.65 (2.67)

Note. Total problems score has a possible range of 0 – 40; All subscales have a possible range of 0 – 10.

Table S3. *Bivariate and Point-Biserial Correlations between SDQ scores and Descriptives*

	Sex of Child	Ethnicity	Age Entered Care	# Placement Providers	Sibling-living Status	Missing Person Record
				**Year 1**		
Total problem score	-.05	-.19**	-.06	.18**	-.14*	.10*
Emotional problems	.07	-0.5	.07	.04	-.13*	.04
Peer problems	-.08	-.21**	.05	.13**	-.20**	.07
Conduct problems	-.10*	-.13**	-.08	.22**	-.05	.16**
Hyperactivity	-.09	-.17**	-.18**	.13**	-.05	.05
Prosocial skills	.14**	.15**	.03	-.10*	.10	-.02
				**Year 2**		
Total problem score	-.05	-.18**	-.03	.19**	-.24**	.13**
Emotional problems	.10*	-.04	.13**	.09	-.17**	.10*
Peer problems	-.05	-.20**	.10*	.10*	-.27**	.08
Conduct problems	-.05	-.09	-.06	.27**	-.17**	.21**
Hyperactivity	-.12**	-.19**	-.22**	.11*	-.11*	.03
Prosocial skills	.10*	.07	-.06	-.10*	.15*	-.12*
				**Year 3**		
Total problem score	-.07	-.17**	-.06	.22**	-.17**	.25**
Emotional problems	.04	-.09	.08	.14**	-.14*	.22**
Peer problems	-.05	-.21**	.08	.18**	-.22**	.18**
Conduct problems	-.05	-.08	-.09	.22**	-.07	.25**
Hyperactivity	-.13*	-.13**	-.20**	.15**	-.09	.12**
Prosocial skills	-.01	.05	-.07	-.10	.09	-.18**

Note. **p* < .05, ***p* < .01. Biological sex of the child was coded as (0) male and (1) female; Ethnicity was coded as (0) White and (1) Minority ethnicity. Total placement providers is

total number of placement providers (for any length of time) over the first 3-years in care; Sibling living status is coded as (0) separated from all siblings and (1) together with at least one sibling; Missing person record is coded as (0) no missing person reports over first 3-years in care and (1) at least 1 missing person report over first 3-years.

Table S4. *Mean total SDQ scores by placement type.*

	M	*SD*
	**Year 1**	
Foster	14.67	7.91
Kinship	12.17	7.86
Residential	15.33	7.64
	**Year 2**	
Foster	14.66	7.55
Kinship	10.70	8.06
Residential	18.05	7.03
	**Year 3**	
Foster	14.01	7.75
Kinship	12.73	7.52
Residential	19.36	6.40

Note. Total SDQ scores can range from 0-40
